# Surface Modification by Plasma Electrolytic Oxidation of Friction Surfacing 4043 Aluminum-Based Alloys Deposited onto Structural S235 Steel Substrate

**DOI:** 10.3390/ma18143302

**Published:** 2025-07-13

**Authors:** Roxana Muntean, Ion-Dragoș Uțu

**Affiliations:** Department of Materials and Manufacturing Engineering, Politehnica University Timișoara, Piața Victoriei 2, 300006 Timișoara, Romania; roxana.muntean@upt.ro

**Keywords:** friction surfacing, aluminum alloys, plasma electrolytic oxidation, wear and corrosion resistance

## Abstract

The friction surfacing (FS) process has emerged over the past few years as a method for joining both similar and dissimilar materials, for volume damage repair of defective components, and for corrosion protection. The possibility to produce a metallic coating by FS, without melting the material, classifies this technique as distinct from other standard methods. This unconventional deposition method is based on the severe plastic deformation that appears on a rotating metallic rod (consumable material) pressed against the substrate under an axial load. The present study aims to investigate the tribological properties and corrosion resistance provided by the aluminum-based FS coatings deposited onto a structural S235 steel substrate and further modified by plasma electrolytic oxidation (PEO). During the PEO treatment, the formation of a ceramic film is enabled, while the hardness, chemical stability, corrosion, and wear resistance of the modified surfaces are considerably increased. The morpho-structural characteristics and chemical composition of the PEO-modified FS coatings are further investigated using scanning electron microscopy combined with energy dispersive spectroscopy analysis and X-ray diffraction. Dry sliding wear testing of the PEO-modified aluminum-based coatings was carried out using a ball-on-disc configuration, while the corrosion resistance was electrochemically evaluated in a 3.5 wt.% NaCl solution. The corrosion rates of the aluminum-based coatings decreased significantly when the PEO treatment was applied, while the wear rate was substantially reduced compared to the untreated aluminum-based coating and steel substrate, respectively.

## 1. Introduction

To ensure sustainable development, environmental conditions intended to boost resource usage efficiency, decrease waste output, and minimize harmful emissions have been intensely discussed. Numerous strategies have been considered in various industries to meet the specific requirements. In the transportation sector, where more than 25% of worldwide greenhouse gas emissions are released [1], significant efforts are directed towards lowering vehicle energy consumption by promoting electric vehicles, improving fuel efficiency, considering alternative fuels, or reducing CO_2_ emissions by adopting new design solutions [2]. Among all the factors that can influence energy consumption and gas emissions, respectively, the compactization/weight reduction of vehicles is regarded as a viable solution. Greater use of lightweight materials (aluminum, magnesium, titanium) is one of the key approaches to lower CO_2_ emissions, notably in the transportation industry [3]. Due to their outstanding properties, including superior corrosion resistance, increased formability, recyclability, and high strength-to-weight ratio, aluminum and its alloys have drawn a lot of attention in this specific manufacturing sector [4]. According to a published report by The Center for Automotive Research [5], lightweight vehicles are considered a top priority for the automotive industry. Mass reduction is a resourceful pathway to better recycling and improvement of vehicle performance [6]. Moreover, the possibility of joining light metals and steel facilitates the production of hybrid structures with significantly enhanced performance for the automotive industry [2]. Including light-metal components in the car body or ship hulls provides a significant weight reduction for vehicles, thus lowering emissions. In this aim, one promising and environment-friendly route is friction surfacing (FS), which is a cladding process for surface coating [7] proposed by Klopstock and Neelands in 1941 [8]. The FS process has emerged over the past few years as a method for joining both similar and dissimilar materials and for volume damage repair of expensive components [9]. FS has been continuously developed over time, with several variants, such as friction stir forming, friction stir deposition, and lateral friction surfacing [10]. This technique is considered a solid-state process, as the coatings are created at temperatures below the melting range of the involved materials [11]. FS, which is related to friction welding, employs the energy generated during the process and creates a layer of plasticized metal without using an external heat source. The possibility to produce a metallic coating without melting the material classifies this technique as distinct from other standard deposition methods [9]. The unconventional deposition route is based on the severe plastic deformation that appears on a rotating metallic rod (consumable material) pressed against the substrate material under an axial load [11]. The metallic rod used in FS is consumed during the process [7]. Since no external heat source is provided, the frictional energy generated exactly at the interface ensures the softening of the consumable material. As a consequence, a solid-state transfer occurs as a continuous shearing layer, formed during the translation at a certain speed along the substrate [12].

The most relevant process parameters of FS are related to the properties of the consumable material (melting temperature, hardness, thermal conductivity); process conditions (surface roughness, thickness, and size of the substrate; atmosphere); as well as other significant process parameters, such as applied axial force, rotational speed, coating speed, and diameter of the consumable rod [13,14]. The appropriate combination of parameters is vital and has a great influence on the coating characteristics, such as the resulting thickness, hardness, surface roughness, and bonding strength. Many investigations have dealt with the impact of the process parameters on the resulting FS coatings. Chandrasekaran et al. [15] investigated the FS of steel, Inconel, aluminum, and titanium rods onto mild steel and aluminum substrates. It was found that nickel-based alloys and steel rods were effectively deposited onto the mild steel substrate, while deposition on the aluminum substrate was only possible under high axial loads. The study concluded that FS can be used as an alternative method for obtaining coatings of dissimilar materials, like bonding steel with aluminum, which is difficult through other fusion-based joining processes, since both metals are metallurgically immiscible with each other. Recently, successful multi-layer FS was reported as a feasible solution for depositing aluminum on steel [16,17] or even aluminum on aluminum [18].

The effects of friction process parameters (rotation speed, coating speed, and feed rate) on the microstructure and properties of aluminum deposition onto steel were intensely investigated [7,12]. It has been generally shown that an increase in the feed rate leads to higher deposition bonding strength and lower surface roughness, while an increase in the coating speed reduces the deposition thickness and bonding strength but improves the surface quality. A higher rotation speed has a negative influence on the deposition width and surface quality. A lower rotational speed affects the temperature developed in the process, resulting in thicker coatings [19]. The typical thickness of the FS coatings generally ranges from 0.5 to 3 mm, depending mainly on the material and diameter of the coating rod [20]. Applying a higher axial load leads to an increase in the bond’s strength, reducing the coating thickness [21].

During the deposition process, several mechanical and metallurgical phenomena were identified: plastic deformation, recrystallization, phase transitions, and the absence of fusion during metal transfer. The maximum temperature achieved during the process is under the melting point of the metallic rod; thus, the occurrence of defects such as porosity, internal stress, and solidification cracking is avoided [14]. The coatings obtained by FS generally display a dense, clean, and fine microstructure [20], a good metallic bond, and a solid diffusion interface [13]. Compared to the conventional fusion-based deposition methods, a limited impact on the substrate and a reduced heat-affected zone is ensured [11,14]. Furthermore, there is no dilution of the coating by the substrate. The FS process is regarded as an environmentally friendly method, since shielding gases or cooling methods are not involved [14]. In addition, a great advantage of the FS process consists of the possibility to join divergent materials, like aluminum and steel, since solid adhesion is achieved by generating high contact stresses [22]. Moreover, both aluminum and steel possess high thermal conductivity, which leads to difficulties in producing a layer under other conditions. Hence, a solid-state bonding process like FS is more suitable for this purpose. The limitations of the FS process refer mainly to the surface quality, which is typically inferior compared to other deposition methods. Furthermore, when aluminum and its alloys are considered for FS, the surface requires additional treatments to overcome their moderate corrosion behavior and wear properties. Despite the unique properties of aluminum, including its capability to form a natural passivation layer, the susceptibility to different kinds of local corrosion, such as stress corrosion cracking, pitting corrosion, intergranular corrosion, and exfoliation corrosion under extreme conditions, is a major challenge [23]. In this case, several modification treatments are usually applied, such as conversion coatings, anodizing, or plasma spray coatings. Among the available options, plasma electrolytic oxidation (PEO) has been proven to be a useful and environmentally friendly surface modification technique, applied especially for lightweight metals and alloys (Al, Mg, Ti, Zr) [24]. Research on PEO coatings has garnered global attention in recent years. Most of this research has focused on optimizing process parameters to create flawless coatings with exceptional resistance to corrosion and wear or on understanding the mechanism of the PEO process and how micro-discharges affect the morphology, structure, and phase composition of coatings. PEO includes several electrochemical, chemical, and thermochemical reactions [25] when the surface to be treated is subjected to high voltages, greater than the dielectric breakdown voltage of the oxide film, in a suitable electrolyte. Consequently, numerous micro-discharges appear on the exposed surface, accompanied by intense gas evolution and the formation of a thick ceramic coating. The generated coating contains elements from both the substrate and the electrolyte solution [26]. Several additives included in the electrolyte, like different ceramic particles, may lead to the formation of composite coatings, where the particles are electrophoretically incorporated in the final coating. By adjusting the polarization conditions, several modes, including direct current, alternating current, or pulsed current, are accessible to produce such coatings [27].

In this regard, the current study aims to investigate the possibility of modifying the surface of an FS-deposited 4043 aluminum-based alloy onto an S235 structural steel substrate by applying a PEO treatment in an alkaline electrolyte with and without additives. The novelty of the study lies in the use of friction surfacing to create an aluminum layer on a structural steel substrate, offering a lightweight and corrosion-resistant alternative for steel components, combined with the application of a PEO treatment, which significantly enhances both corrosion and wear resistance. During the treatment, direct and pulsed current mode is employed. The ceramic films created on aluminum-based coatings are further characterized in terms of microstructure, adhesion, surface roughness, wear, and corrosion behavior. The proposed hybrid structures may be suitable for structural components requiring corrosion- and wear-resistant outer layers on steel substrates in the automotive, marine, and aerospace sectors. The findings contribute to the advancement of hybrid surface modification strategies for steel components used in aggressive environments.

## 2. Materials and Methods

Commercially available S235 structural steel (ArcelorMittal Europe, Luxembourg) was used as substrate material for friction surfacing of the aluminum-based alloy, 4043 series (Lion Metal, Shanghai, China), known for its good corrosion resistance and weldability. The S235 structural steel was selected as a substrate due to its widespread use in construction, transportation, and general engineering applications, where cost-effective materials with adequate mechanical strength are required. The microstructure of the S235 steel, confirmed via optical microscopy, consists primarily of equiaxed ferrite grains with interspersed lamellar pearlite colonies, typical of a ferrite–pearlite structure. This microstructure confers the material’s formability and weldability, making it a suitable substrate for friction surfacing applications. Furthermore, using the S235 allows for the evaluation of aluminum coatings applied via FS on low-carbon steel, simulating real-world scenarios where lightweight, corrosion-resistant aluminum layers are added to structural steel components. This combination offers the potential for improved performance in corrosive environments without compromising the mechanical integrity of the underlying steel. The quality and chemical composition of the steel substrate and the aluminum alloy, respectively, were analyzed using an optical emission spectrometer (OES) (Thermo Electron Corporation Genesys, Thermo Fisher Scientific, Waltham, MA, USA), and the weight concentration values along with their associated uncertainties are presented in Table 1.

**Figure 1:**
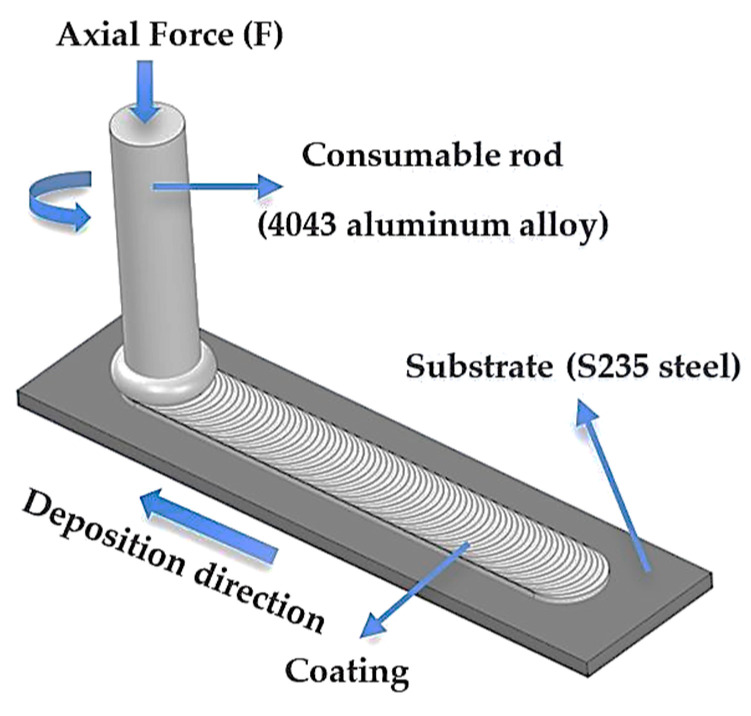
Schematic representation of the FS process of aluminum 4043 alloys on S235 steel substrate.

Before applying the FS process, the substrate was cut into 40 mm × 20 mm × 5 mm coupons, and the surface was ground with abrasive paper (80 SiC paper grit) (Struers Detroit, OH, USA), to remove the oxides, then degreased with ethanol (Carl Roth, GmbH + Co. KG, Karlsruhe, Germany) and fixed in a bench vise. For the FS process, experimental equipment based on a vertical drilling machine (Alzmetall AX 2/S Altenmarkt, Germany) was adapted, where the aluminum alloy, employed as a consumable rod with a 19 mm diameter, was cast formed in the laboratory from the commercial 4043 aluminum based alloy. The Al-based rod was mounted in a holder and attached to the FS system, according to the schematic representation shown in Figure 1.

The FS parameters applied for generating compact and qualitative coatings with improved adhesion and strong bonding are a nominal contact pressure of 7 MPa, feed rate of 2 mm s^−1^, and spindle rate of 2000 rpm. These parameters were maintained constant for all the specimens of the current study, which were afterwards subjected to the PEO treatment. The thickness of the aluminum-based coating obtained after one single pass of the consumable rod was around 1 mm. A common defect observed on the FS coatings is the unbounded region found at the edge of the deposits, due to the deformation of the consumable rod in contact with the substrate [28]. This aspect remains a serious problem in FS technology that needs to be solved. It has been demonstrated that an inferior diameter of the consumable rod leads to defects in the coating and at the interface, due to a lower surface contact area [12]. The resulting FS samples were further processed by cutting and grinding with abrasive paper up to a roughness of *R_a_* = 0.4.

**Figure 2:**
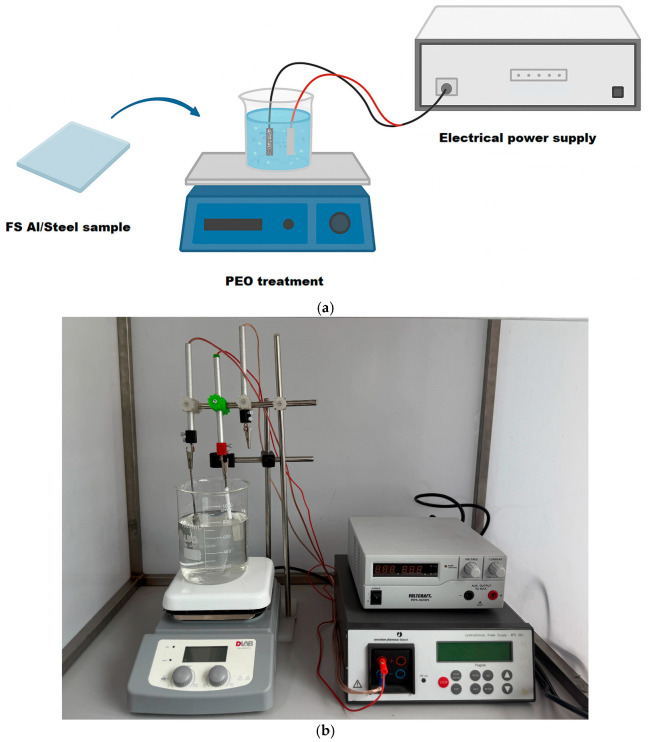
(**a**) Simplified schematic representation of the PEO process; (**b**) experimental setup.

Subsequently, the PEO surface modification process was carried out in a two-electrode cell, according to Figure 2, where the FS aluminum-based coating was positioned at the anode and a rectangular stainless-steel plate (type 304) (ArcelorMittal Europe, Luxembourg) was placed at the cathode. To avoid the intense oxidation of the substrate material, the back side of the samples was sealed with epoxy resin (Struers Detroit, OH, USA), keeping the exposed surface only on the aluminum-coated side (8 cm^2^). The first electrolyte used for the PEO treatment was prepared using distilled water by mixing 4 g L^−1^ KOH and 20 g L^−1^ Na_2_SiO_3_ at a pH of 12. Additionally, a second electrolyte was prepared by mixing 4 g L^−1^ KOH and 3 g L^−1^ Na_2_WO_4_·2H_2_O in distilled water, at pH 12. Two different polarization modes were applied for each type of electrolyte, with the aid of an electrical power supply (Amersham Pharmacia Biotech, Inc. Electrophoresis Power Supply, EPS 601, Buckinghamshire, United Kingdom), coupled with an autotransformer and a bridge rectifier, one using direct current (DC) and the second one with pulsed current (PC). In the case of PC treatment, a unipolar current was applied with the aid of an electrical relay, setting a duty cycle (*d.c.*) of 20%, at a frequency of 0.2 Hz. The PEO process was conducted in galvanostatic mode, keeping the current density constant at 30 mA cm^−2^. The most important parameters and treatment settings, presented in Table 2, were chosen to ensure the conditions for plasma generation, which is known to occur between 300–400 V in silicate-based electrolytes. Consequently, the current density was adjusted to initiate the plasma conditions but also to keep the power consumption as low as possible and to reduce the operational costs. As the oxide layer becomes thicker, higher voltage is required to cause dielectric breakdown and spark formation. However, softer or more stable discharges reached at lower voltages result in smoother, denser, and less porous coatings. The treatment time controls the thickness of the oxide coating, but it was observed that longer times may lead to cracking. The current regime (direct current or pulsed current) controls the porosity and the morphology of the coatings.

**Figure 3:**
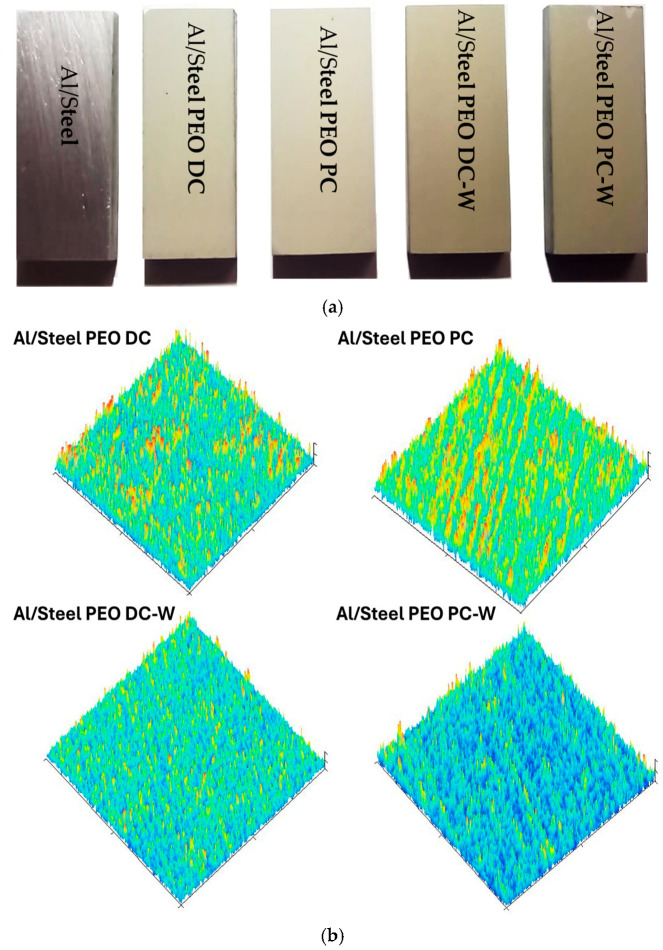
(**a**) Macrographic image of the Al/Steel samples obtained using different sets of parameters and (**b**) their corresponding 3D surface profilograms.

A short name was assigned for each sample, indicating the polarization mode and the type of electrolyte. The most representative samples treated using different sets of parameters are shown in the macrographic image from Figure 3a. As observed, the PEO treatment changed the bright metallic surface of the aluminum to a porous grey ceramic film, characteristic of a PEO-modified surface. The samples treated in the tungsten-containing electrolyte present a slightly darker surface, compared to the samples obtained from the silicate-based solution. To further investigate the surface morphology beyond the optical observations, 3D surface profilograms were acquired for all PEO-treated samples and are presented in Figure 3b. These profilograms provide a topographical visualization of the surface structure, highlighting differences in surface roughness and feature distribution induced by the various PEO treatment conditions. The Al/Steel PEO PC sample exhibited the highest surface roughness, with pronounced peak structures and a more heterogeneous texture compared to the DC-treated one. In contrast, the addition of tungsten in the electrolyte (Al/Steel PEO DC-W and Al/Steel PEO PC-W) led to smoother surfaces with reduced peak intensity and more uniform profiles. These observations align with the visual contrasts observed in the macrographic images (Figure 3a) and offer an insight into how the current regime and electrolyte composition affect the surface topography. All profilograms were obtained under the same scanning parameters, and the height scales were kept consistent to allow for direct comparison among the samples.

The morphology and structure of the PEO-modified aluminum-based samples were analyzed using a Tescan Vega 3 scanning electron microscope (SEM) (Tescan Group a.s., Brno, Czech Republic) equipped with an energy-dispersive spectroscopy analyzer (EDAX, Ametek Inc. Mahwah, NJ, USA) for chemical composition analysis and an optical microscope (Leica DM 2700M, Wetzlar, Germany). To examine the cross-section of the samples, the specimens were metallographically prepared according to the ASTM E3-11 standard [29]. The phase evaluation was realized with the aid of an X-ray Diffractometer (XRD) (PANalytical X’Pert Pro Powder Malvern Panalytical, Malvern, UK), with Cu-Kα radiation (λ = 1.54), and the measurements were carried out between 20° and 100° 2ϴ-angles, with a step size of 0.033, at 45 kV and 30 mA. The roughness of the samples was evaluated before and after the PEO treatment with a Mitutoyo SJ-201 portable surface tester (Mitutoyo, Aurora, IL, USA). The adhesion of the aluminum-based coating on the steel substrate was estimated by applying Brinell hardness measurements (Universal Hardness Tester Nexus 601, Innovatest, Maastricht, The Netherlands) while gradually increasing the loads at the interface. The corrosion resistance of PEO-modified FS aluminum-based samples was investigated using open-circuit potential (OCP) measurements and potentiodynamic polarization. Each type of sample was tested three times to ensure reproducibility of the results. The obtained corrosion resistance values were evaluated and compared to the non-modified FS aluminum-based coatings, with a pure aluminum sample as a reference. The electrochemical measurements were carried out in a three-electrode corrosion cell connected to an Autolab PGSTAT 302 N potentiostat/galvanostat (Metrohm, Herisau, Switzerland). The working electrode consisted of the Al-based samples (as-deposited or PEO-modified), with an exposed surface area of 1 cm^2^; the reference was an Ag/AgCl (3 M NaCl) electrode placed in the proximity of the working electrode with the aid of a Luggin capillary, and the counter electrode was a Pt mesh. The corrosion measurements were recorded in 3.5 wt.% NaCl solution at normal temperature, with scan rate of 0.16 mV s^−1^. The most important corrosion parameters, *E_corr_*, *i_corr_*, and *v_corr,_* were estimated from the polarization plots using the Tafel extrapolation method, in the linear region of the anodic and cathodic branches.

The dry sliding wear behavior of the PEO-modified FS aluminum-based samples was evaluated using the ball-on-disc method according to the ASTM standard G99 [30]. The measurements were carried out with the aid of a tribometer (TR-20, Ducom-Materials Characterisation Systems, Groningen, The Netherlands), under dry sliding conditions and ambient temperature, with a normal load of 10 N, sliding rate of 100 rot min^−1^, total time t = 100 min, and a total sliding distance of 260 m. The static partner was an Al_2_O_3_ ball of 6 mm diameter. During the measurements, the variation of the coefficient of friction (COF) with the distance was automatically recorded. The profile of the wear track was analyzed using a confocal laser scanning microscope (CLSM, Keyence VK-X 3D, Keyence International, Mechelen, Belgium), and the material loss and wear rate were estimated. For each type of sample, three measurements were performed to ensure the reproducibility of the results.

## 3. Results and Discussion

### 3.1. Morphological, Chemical, and Phase Composition of the FS Al-Based and PEO-Treated Coatings

**Figure 4:**
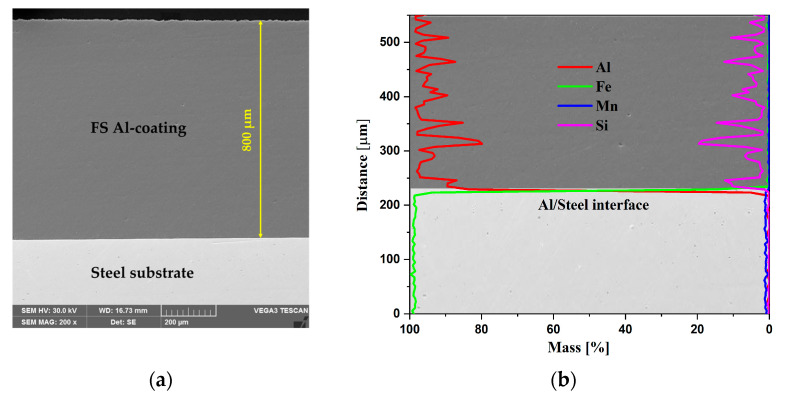
Cross-section SEM micrograph of the Al/Steel sample (**a**); the corresponding EDX line scanning at the interface, 200 µm below and 350 µm above (**b**).

The cross-section micrograph of the FS Al/Steel sample presented in Figure 4a reveals a clean and defect-free bond along the interface. It is important to note that the samples were not etched before imaging to preserve the original interface characteristics. No gaps between the aluminum coating and the steel substrate, or any cracks and pores in the coating, can be observed. The thickness of the Al coatings is found to be between 800 and 1000 µm, measured after the surface was processed with SiC paper, to achieve a roughness of around *R_a_* = 0.4. The EDX line scanning presented in Figure 4b was performed starting from 200 µm below the Al/Steel substrate interface to 350 µm above. The main elements from the substrate (Fe and Mn) are highlighted, while Al and Si show up only in the coating. The elemental distribution across the Al/Steel interface reveals important insights into the metallurgical bonding mechanisms during aluminum friction surfacing on S235 steel. The sharp transition between high Fe content in the steel substrate and high Al content in the deposited layer indicates a well-defined interface with minimal elemental interdiffusion. This suggests that bonding primarily occurred through a solid-state joining mechanism, facilitated by severe plastic deformation and frictional heat rather than melting, since the maximum temperatures achieved are in the range of 400–450 °C [31]. The absence of a significant diffusion zone or thick intermetallic layer implies that the process parameters were optimized to avoid excessive thermal exposure, which is critical in preventing the formation of brittle Fe-Al intermetallic compounds (Fe_2_Al_5_, FeAl_3_), which can compromise joint integrity. Overall, microstructural evidence supports a strong metallurgical bond dominated by mechanical interlocking and limited atomic interaction, characteristic of high-quality friction surfacing.

**Figure 5:**
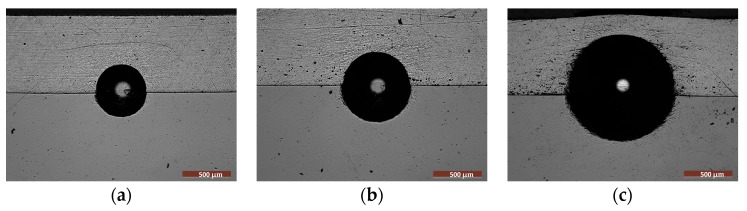
The adhesion of the aluminum-based coating on the steel substrate, estimated with the Brinell hardness method, by applying different loadings for 15 s: (**a**) 15.625 N; (**b**) 30 N; (**c**) 62.5 N.

In this study, Brinell hardness testing was used to provide indirect insight into the adhesive properties of the FS Al-based coatings. Although Brinell hardness testing is not regarded as a standard practice to directly determine the adhesion of a coating to the substrate, in the current work, it was applied at the interface using different loads to detect interface failure after applying a specific load. The method can provide qualitative information about the coating-substrate adhesion only when coupled with visual inspection, usually microscopy. The occurrence of cracks, spalling, delamination, or coating chipping at the indent edges is expected. The method does not offer a numerical adhesion strength but qualitatively ranks adhesion based on the mechanical damage response. It may be suitable for hard and brittle coatings, like oxides, nitrides, and even PEO-generated coatings. However, the indentations at different loads did not determine the detachment or cracks propagated along the interface of the Al coatings, even when the highest load was applied, as observed in the optical micrographs illustrated in Figure 5.

**Figure 6:**
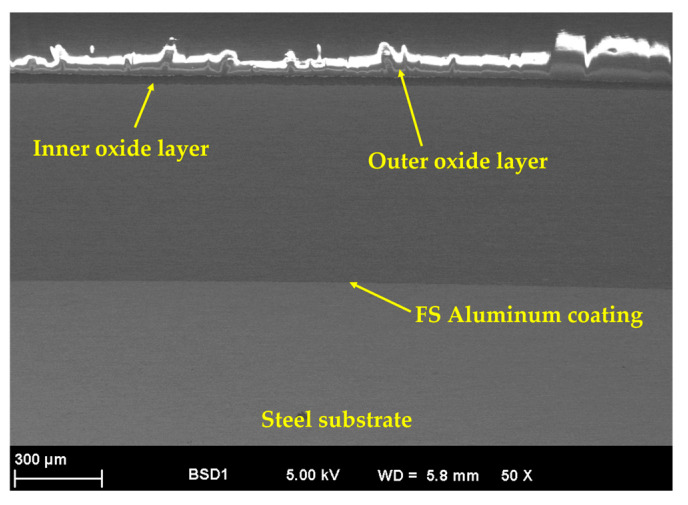
Cross-section SEM micrograph, specifically for an Al/Steel PEO-treated sample.

**Figure 7:**
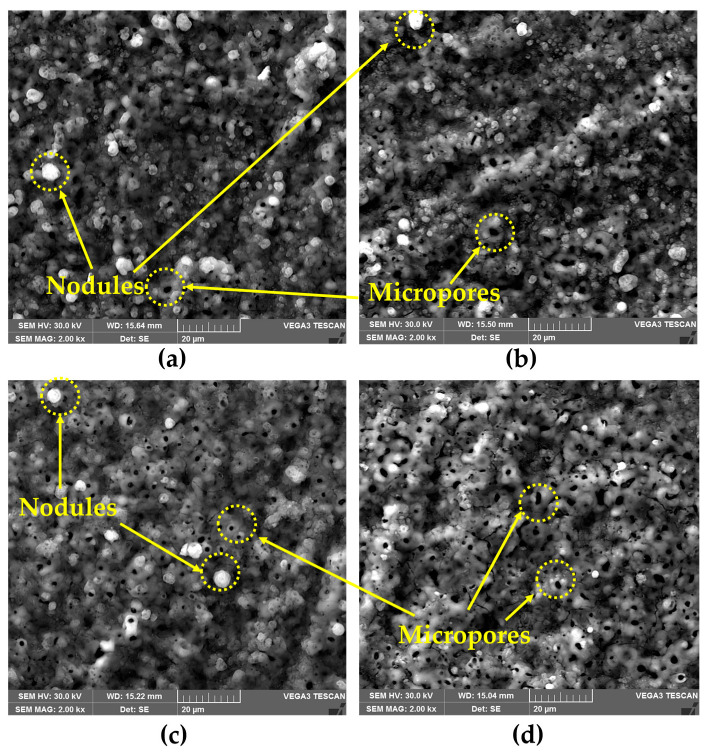
Surface SEM micrograph of the (**a**) Al/Steel PEO DC; (**b**) Al/Steel PEO PC; (**c**) Al/Steel PEO DC-W; and (**d**) Al/Steel PEO PC-W.

Figure 6 presents the cross-section SEM micrograph of the Al/Steel PEO-treated samples, revealing the microstructure of the aluminum oxide layer formed during the plasma treatment. Since the treatment time was maintained constantly for all samples, the appearance of the oxide layer is similar. The passive layer has a relatively uniform thickness of around 50 µm and exhibits a microporous structure, formed by two distinct layers, as depicted in the SEM micrograph. Initially, the plasma discharge is less intense and creates a denser structure in the form of a compact oxide inner layer (dark grey region). When this oxide layer reaches a specific thickness, the current flow is partially disrupted, and the plasma discharge becomes more intense, due to the ionization of the gas bubbles released at the electrode. Once the dielectric breakdown voltage threshold of the previously formed oxide layer is exceeded [32], high temperatures are reached, determining a localized melting of the metal substrate. Several electrochemical reactions occur, and the reaction products are expelled from the plasma discharge channels and further deposited around the pores, forming the outer oxide layer. As a result, different micro-sized pores, like craters and shallows, are distributed all over the surface of the PEO-treated samples, according to Figure 7a–d.

**Figure 8:**
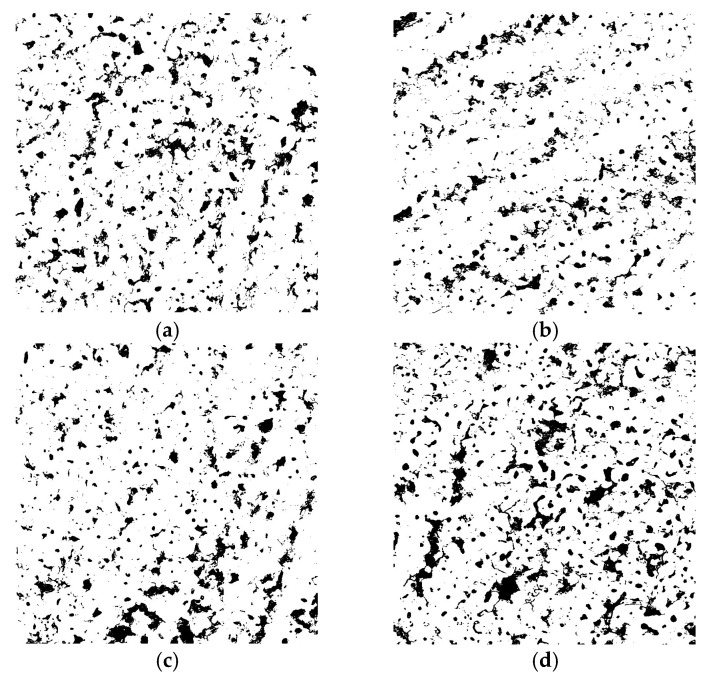
Porosity fraction of the PEO-treated samples estimated from SEM micrographs using ImageJ software: (**a**) Al/Steel PEO DC (10.68%); (**b**) Al/Steel PEO PC (9.22%); (**c**) Al/Steel PEO DC-W (10.49%); and (**d**) Al/Steel PEO PC-W (13.87%).

**Figure 9:**
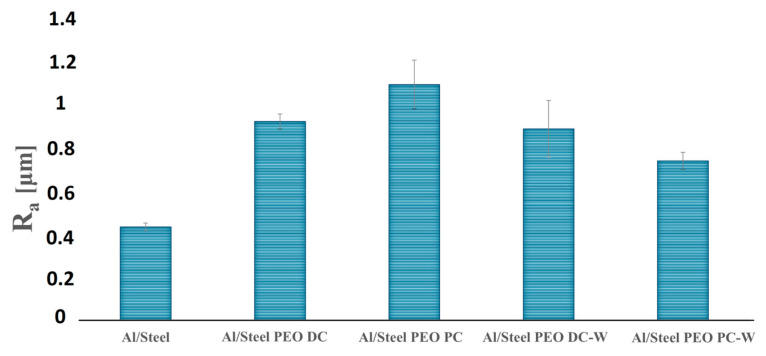
*R_a_* surface roughness values for the Al/Steel and PEO-treated samples. The corresponding average *Rz* values are Al/Steel (2.25); Al/Steel PEO DC (4.66); Al/Steel PEO PC (5.57); Al/Steel PEO DC-W (4.78); and Al/Steel PEO PC-W (4.05).

In addition, the precipitation of the reaction products transferred through the passive layer leads to the formation of hydroxides, oxyhydroxides, or even oxides. The volcano-crater-like structure, typically observed for the PEO-treated coatings, appears due to the plasma treatment conditions and the intense gas evolution that takes place during the oxidation process. The high electron density that promotes micro-discharges leads to the formation of channels in the oxide layer. The average porosity estimated from the SEM images using ImageJ software is between 9 and 14%, according to Figure 8, with a predominant pore diameter range of 2–5 µm. Analyzing the surface micrographs, it appears that the structure is similar for all the specimens, regardless of the electrolyte type or the current regime. In the case of the samples treated using PC, a higher porosity was observed compared to the samples obtained by DC, where the structure was more compact. However, the mitigation of intrinsic porosity is a major challenge in the actual PEO technology. One of the most effective techniques for reducing porosity in PEO coatings is the electrolyte formulation. Consequently, the addition of W in the electrolyte (in the form of Na_2_WO_4_·2H_2_O) determined a pore refinement compared to the Si-based electrolyte. In agreement with the porosity appearance, the surface roughness measurements revealed slightly lower average *R_a_* values for the samples obtained from the W-based electrolyte, according to Figure 9. This may be attributed to a lower release of gaseous products during oxidation, correlated with the inclusion of W in the matrix of the oxide layer, which requires additional electric charge. The roughness of the samples was also influenced by the current regime. PC usually results in coatings with lower roughness and delivers shorter plasma discharge periods compared to DC, reducing in this way the occurrence of large, destructive channels.

**Figure 10:**
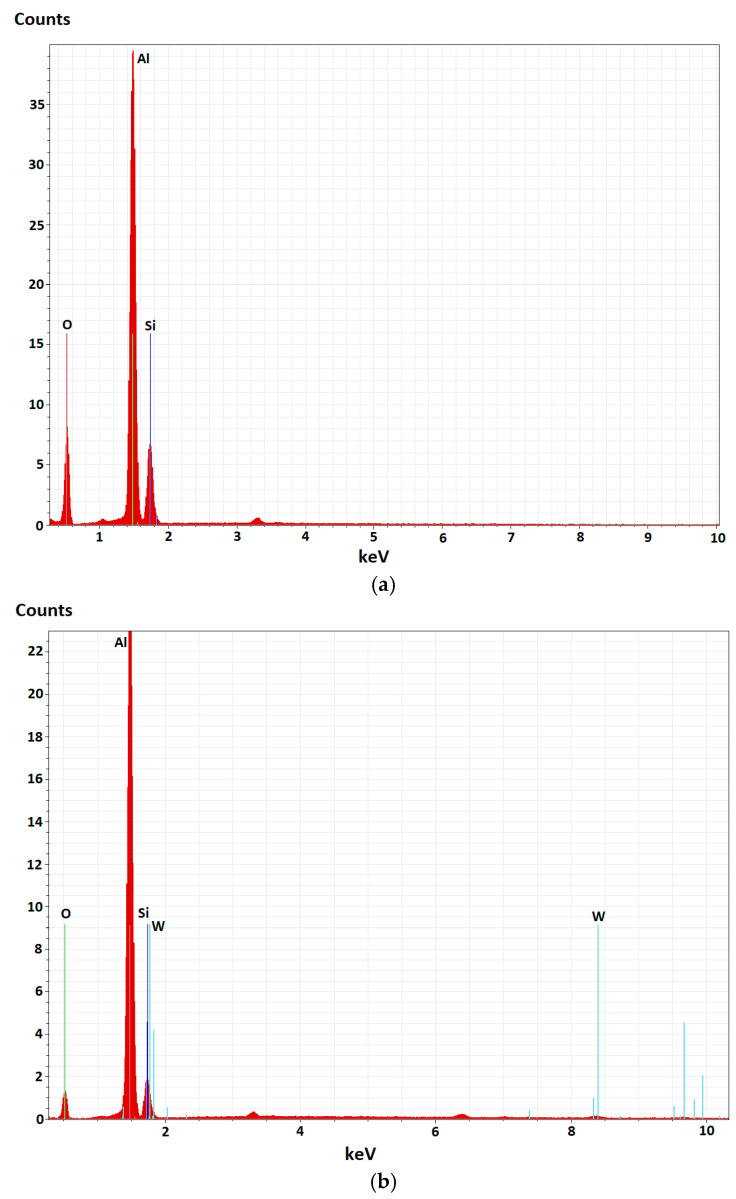
EDX analysis of (**a**) Al/Steel PEO DC and (**b**) Al/Steel PEO DC-W.

The EDX analysis depicted in Figure 10, performed on the PEO-treated surfaces, shows the elemental content of the samples. The spectra confirm the presence of Al as the main component of the coatings but also reveal additional chemical elements from the oxide layer that were incorporated from the electrolyte composition, like Si and W, demonstrating the successful integration of such constituents in the coating’s matrix through the plasma-chemical reactions that took place. During the PEO process, the electrolytes additionally provide oxygen, which reacts with the molten metal substrate, resulting in the formation of ceramic oxides, in this case, mainly Al_2_O_3_.

**Figure 11:**
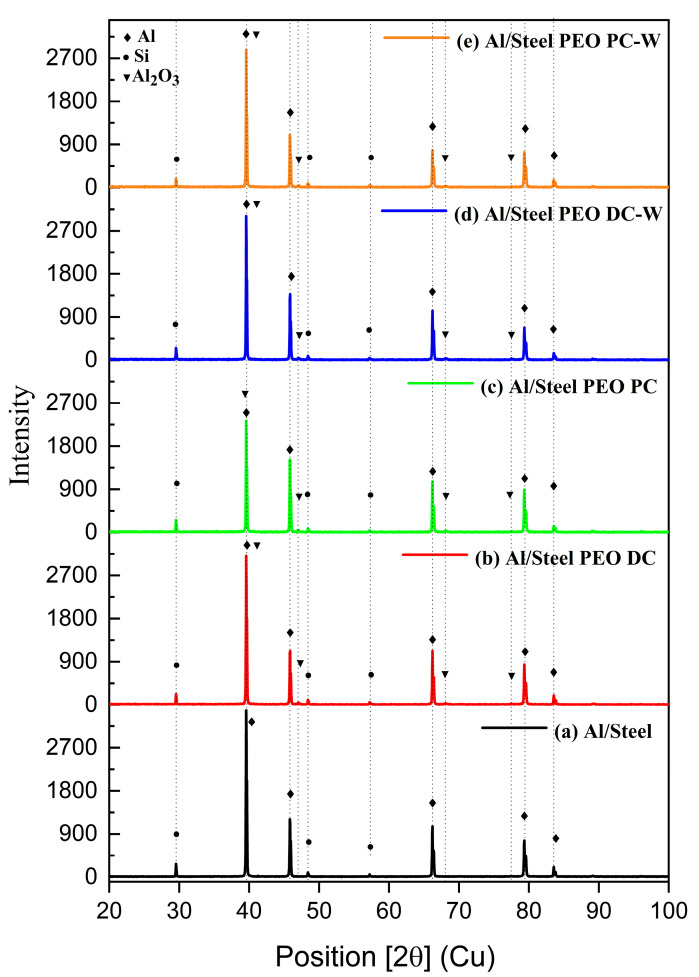
XRD diffraction patterns of (**a**) Al/Steel; (**b**) Al/Steel PEO DC; (**c**) Al/Steel PEO PC; (**d**) Al/Steel PEO DC-W; and (**e**) Al/Steel PEO PC-W.

The presence of Al_2_O_3_ phases is additionally highlighted in the XRD patterns presented in Figure 11 for all plasma-treated specimens. It should be noted that the XRD patterns primarily show the aluminum phases, as the coatings are approximately 800 µm thick. This thickness is sufficient to absorb the incident X-rays and prevent them from reaching the steel substrate, thus explaining the absence of steel-related diffraction peaks.

The XRD investigations reveal the most significant phases present in the composition of the aluminum-based FS coatings. All the identified peaks confirm the crystallinity of the coatings. The diffraction peaks for Al and Si are found for all the samples, and they occur from the FS coating, due to the penetration of the X-rays through the oxide layer [33], while for the specimens exposed to plasma, the Al_2_O_3_ phase was additionally observed. The presence of W-based phases is not highlighted in the diffraction patterns of the samples treated in tungsten-containing electrolytes, since the lower amount of W is under the detection limit of the method, and the W-formed phases during the plasma treatment are mostly integrated in the Al_2_O_3_ coatings.

### 3.2. Corrosion Resistance of the FS Al-Based and PEO-Treated Coatings

**Figure 12:**
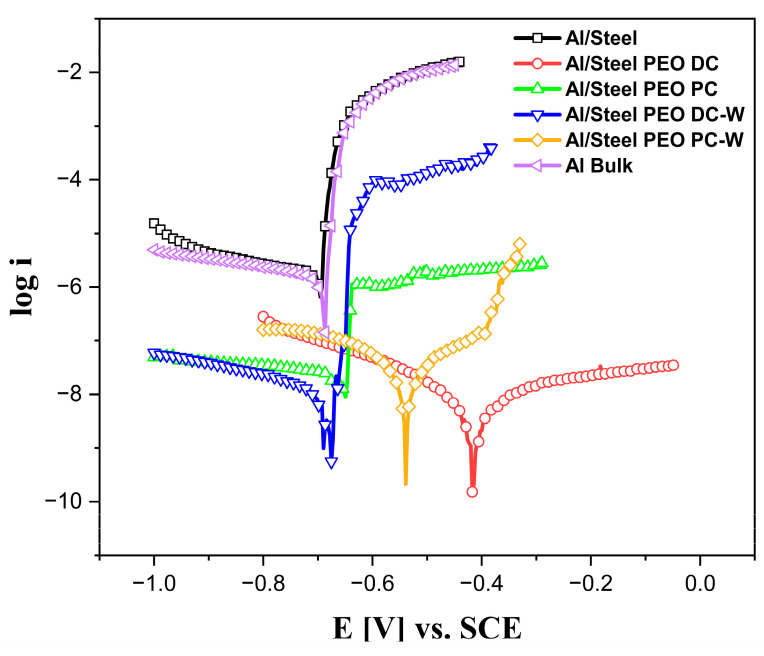
Potentiodynamic polarization curves in semi-logarithmic form measured in 3.5 wt.% NaCl solution at room temperature and 0.16 mV s^−1^ scan rate.

The corrosion resistance of the Al-based coatings was evaluated in 3.5 wt.% NaCl solution, at room temperature, by potentiodynamic polarization. Before each measurement, the OCP values were recorded for 30 min to determine the potential range for each sample. The semi-logarithmic form of the polarization curves is illustrated in Figure 12. The Tafel extrapolation method was applied to estimate the main corrosion parameters, like corrosion current density (*i_corr_*), corrosion potential (*E_corr_*), and corrosion rate (*Corr rate*), and the values are listed in Table 3.

Examining the corrosion parameters and comparing the polarization curves depicted in Figure 12, it can be concluded that the untreated Al/Steel sample behaves identically to the Al bulk reference in terms of corrosion resistance. Both samples present a similar corrosion current density and corrosion potential in the described testing conditions. Generally, aluminum forms a native oxide that offers surface passivation, but it is still prone to localized corrosion, especially in chloride environments. Comparable corrosion behavior was obtained for aluminum in 3.5 wt.% NaCl electrolytes in similar testing conditions [33,34]. For all the PEO-treated Al-based coatings, a significant decrease in the corrosion current density was observed, suggesting an improvement in terms of corrosion resistance. This aspect is attributed to the formation of a dense, crystalline oxide layer composed mainly of γ-Al_2_O_3_ and α-Al_2_O_3_, as evidenced by the XRD measurements. These ceramic phases are chemically stable and minimally soluble, and they substantially reduce electron and ion transport across the coating. It is considered that the oxide film formed anodically on aluminum is a tenacious layer with high effectiveness in providing corrosion protection. The oxide film behaves as a dielectric layer and acts as a physical barrier to further reactions between the metallic aluminum and the environment. Aluminum oxides have low ionic conductivity, so they inhibit both electron flow and ion migration, key steps in electrochemical corrosion reactions. In addition, the PEO process, particularly when performed in direct mode, yields a coating with reduced porosity and improved microstructural integrity, further impeding electrolyte penetration. The reductions of the current density values up to 2–3 orders of magnitude compared to bare aluminum (10^−5^–10^−4^ A cm^−2^) are consistent with the robust PEO performance observed by other research groups [35]. The observed differences in the current densities between different samples arise from the distinct electrochemical behavior of each surface condition. In particular, the higher currents in some coated samples may reflect differences in coating porosity, roughness, oxide composition, or incorporation of other ceramic phases. However, for some of the PEO-treated samples, the *E_corr_* remains similar to the untreated samples due to the porous structure of the oxide layer generated during plasma, which allows the electrolyte to penetrate rapidly into the layer, reaching the untreated substrate. An important positive shift is noticed in the case of Al/Steel PEO DC, indicating more noble behavior, likely due to the compact oxide layer formed during the PEO process. In the anodic region of the polarization curves, pitting peaks indicate the breakdown of the passive layer and the onset of localized corrosion. The bare Al/Steel and Al Bulk samples exhibit sharp increases in current density, characteristic of pitting corrosion in a NaCl environment. In contrast, samples treated with PEO show significantly reduced or absent pitting peaks. Particularly, the Al/Steel PEO DC-W and Al/Steel PEO PC-W samples demonstrate the best performance in this regard. Incorporating tungsten into the PEO-treated coatings significantly enhances corrosion resistance to pitting. W-based phases improve the coating’s density by filling the pores and cracks, reducing pathways for chloride ions. Their chemical stability and low solubility in NaCl environments contribute to a more robust passive layer. Additionally, W-based particles promote electrochemical stability and may support localized re-passivation. As a result, the PEO samples containing W show minimal anodic current and no pitting peaks, indicating excellent protection against pitting corrosion in aggressive chloride media.

### 3.3. Wear Behavior of the FS Al-Based and PEO-Treated Coatings

**Figure 13:**
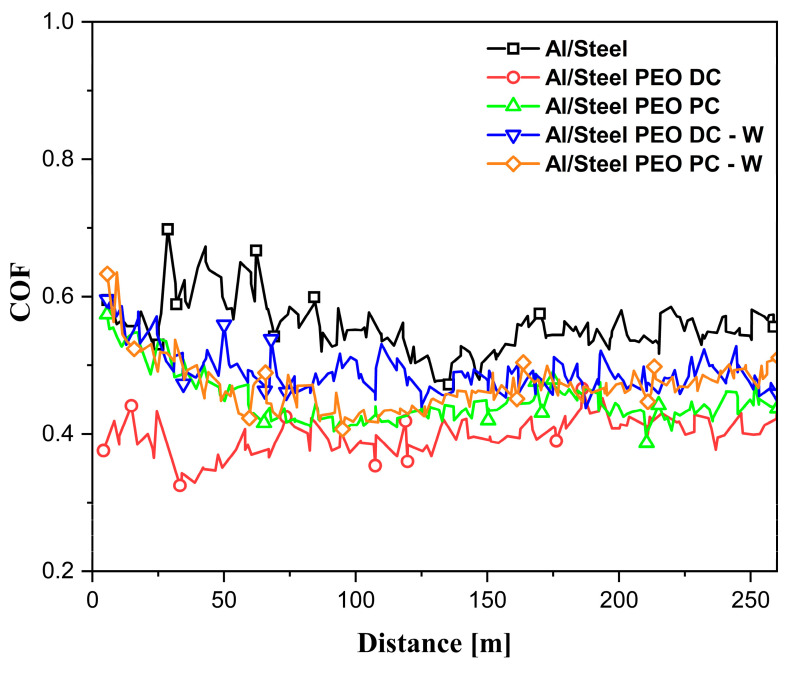
The variation of the coefficient of friction (COF) with the traveled distance.

**Figure 14:**
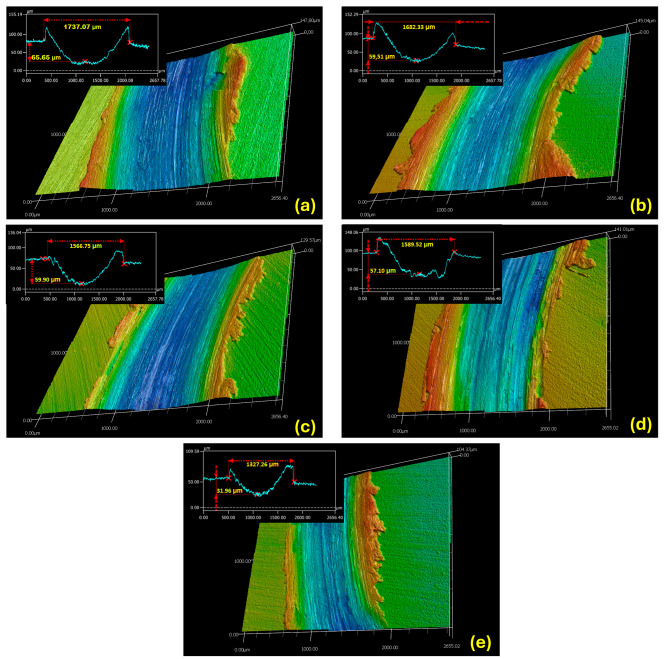
CLSM micrographs of the surface topography of the wear tracks for the Al-based samples: (**a**) Al/Steel; (**b**) Al/Steel PEO DC; (**c**) Al/Steel PEO PC; (**d**) Al/Steel PEO DC-W; and (**e**) Al/Steel PEO PC-W.

**Figure 15:**
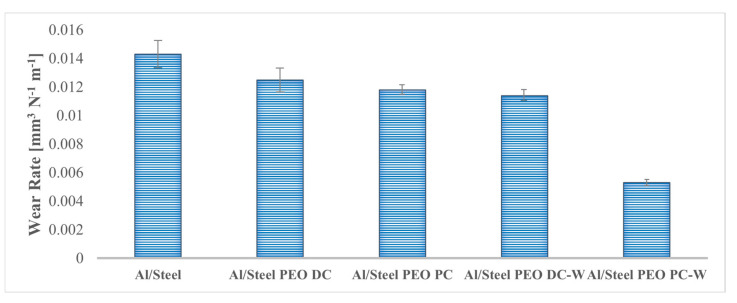
Estimated wear rates for the Al-based samples, with and without PEO treatment.

The wear behavior of the FS aluminum coatings and PEO-treated coatings is studied by conducting dry sliding tests, according to the ball-on-disc method. During the measurements, the coefficient of friction (COF) variation is plotted against the sliding distance, as shown in Figure 13. The maximum, minimum, and average values of the COF are listed in Table 4. During the wear tests, the COF of the untreated Al/Steel sample shows the highest and most fluctuating behavior, with values peaking at nearly 0.9 and averaging around 0.6. This instability may be attributed to the low hardness of the untreated aluminum substrate, which undergoes severe adhesive wear with the highest width of the wear track, due to the ploughing and peeling-off effect, demonstrating poor wear resistance under the given sliding conditions. For the PEO-treated Al samples, the COF considerably decreases and is more consistent during the measurement, due to the presence of the Al_2_O_3_ layer, which provides a higher wear resistance in the testing conditions. The Al/Steel PEO DC exhibits the lowest and most consistent COF, stabilizing around 0.4 throughout the test. The minimal fluctuations of COF imply a consistent wear mechanism and a protective surface. The variation of COF is correlated with the CLSM micrographs, presented in Figure 14, acquired on a section of the wear track for each tested sample. As observed, the Al/Steel coating against the Al_2_O_3_ ball presents severe adhesive wear, due to aluminum’s low hardness and high ductility. The wear track reveals plastic deformation and even aluminum transfer to the counterbody. The samples treated in PEO show a clear improvement in wear resistance, and the wear mechanism shifts to abrasive with brittle wear, typical for ceramic coatings. The coatings’ integrity is mostly preserved, since the oxides act as protective barriers. From the profile of the wear tracks, the specific wear rate was calculated and is comparatively presented in Figure 15. In the wear track analysis, both the volume of material removed (groove) and the volume of displaced material (pile-up) were quantified from the cross-sectional profilograms in Figure 14. This dual approach provides a more comprehensive assessment of wear behavior, particularly for ductile materials where surface deformation contributes to material redistribution. The wear volume was calculated as the area below the original surface level, while the pile-up was determined from the area above this level on both sides of the wear track. Including both aspects allows for a more accurate evaluation of the wear mechanism and the total extent of surface damage. The wear rate provides a normalized measure of wear performance, useful for comparing the coatings under identical testing conditions. The best tribological performance under the described testing conditions was observed for the Al/Steel PEO PC-W, which also presents the lowest wear rate among the treated samples.

Specific wear rate (*W_s_*) is defined by the volume difference of the material after wear (V), the applied normal load (*L*), and the sliding distance (*D*), as follows:

(1) $$W_{s}=\frac{∆V}{L\cdot D}$$

## 4. Conclusions

In this study, the possibility of modifying the surface of the FS aluminum-based alloy deposited onto an S235 steel substrate by applying a PEO treatment in an alkaline environment was investigated.

- The FS Al-based coatings presented good adhesion to the steel substrate, showing a clean interface with no gaps, cracks, or pores in the SEM cross-section micrographs.
- The PEO process was applied successfully to the aluminum surface from two different electrolytes, and the results showed that the PEO is an effective surface treatment, particularly for corrosion resistance enhancement in chloride-rich environments.
- The improvements were associated with the ability of aluminum to form a thick, adherent, and protective oxide layer during the plasma treatment, which minimized the electrochemical degradation.
- The PEO treatments shifted the corrosion potential of the aluminum surface to more noble values and drastically reduced corrosion current density, indicating lower corrosion rates and improved long-term performance.
- The Al_2_O_3_ layer formed during the PEO treatment brought additional benefits in terms of wear resistance.
- The Al/Steel PEO DC sample performed the best, in terms of corrosion and wear resistance, providing the lowest corrosion current densities and the most stable behavior, attributed to the dense and hard Al_2_O_3_ layer.
- The improvements in the wear resistance were evidenced by the lower and more stable coefficient of friction across all the PEO-treated samples.

## Figures and Tables

**Table 1:** The chemical composition of the substrate and the aluminum alloy.

Material	Chemical Composition (wt.%)								
S235 steel	**C**	**Mn**	**P**	**S**	**N**	**Cu**		**Fe**	
	0.16 ± 0.005	1.23 ± 0.01	0.014 ± 0.001	0.008 ± 0.001	0.01 ± 0.002	0.5 ± 0.05		Bal.	
4043 aluminum-based alloy	**Si**	**Fe**	**Cu**	**Mn**	**Mg**	**Zn**	**Others**		**Al**
	4.5 ± 1	0.35 ± 0.05	0.03 ± 0.02	0.03 ± 0.02	0.03 ± 0.02	0.007 ± 0.003	0.12 ± 0.03		Bal.

**Table 2:** PEO treatment conditions for obtaining the FS Al/Steel-modified samples.

Sample	Treatment Conditions			
	Current Density [mA cm^−2^] /Voltage [V]	Total Time [s]	d.c. [%]	Electrolyte
Al/Steel PEO DC	30 mA cm^−2^ /max 370 V	1800	-	4 g L^−1^ KOH + 20 g L^−1^ Na_2_SiO_3_
Al/Steel PEO PC		7200	20	
Al/Steel PEO DC-W		1800	-	4 g L^−1^ KOH + 3 g L^−1^ Na_2_WO_4_·2H_2_O
Al/Steel PEO PC-W		7200	20	

**Table 3:** Corrosion parameters estimated from the Tafel extrapolation of the plots presented in Figure 12 for the Al-based samples with and without PEO treatment.

Scheme 106	OCP [V]	*i_corr_*·10^6^ [A cm^−2^]	*E_corr_* [V]	*Corr Rate* 10^3^ [mm per year]
Al/Steel	−0.68 ± 0.05	1.66	−0.69	19
Al/Steel PEO DC	−0.43 ± 0.02	0.0075	−0.41	0.086
Al/Steel PEO PC	−0.64 ± 0.07	0.0265	−0.66	0.31
Al/Steel PEO DC-W	−0.66 ± 0.08	0.0115	−0.68	0.13
Al/Steel PEO PC-W	−0.55 ± 0.03	0.0204	−0.53	0.23
Al Bulk	−0.68 ± 0.05	1.57	−0.69	18

**Table 4:** Values of the recorded COF presented in Figure 13 for the Al-based samples.

Sample	*μ_min_*	*μ_average_*	*μ_max_*
Al/Steel	0.462	0.560	0.938
Al/Steel PEO DC	0.324	0.394	0.466
Al/Steel PEO PC	0.387	0.452	0.758
Al/Steel PEO DC-W	0.437	0.493	0.673
Al/Steel PEO PC-W	0.404	0.471	0.705

## Data Availability

The original contributions presented in this study are included in the article. Further inquiries can be directed to the corresponding author.
